# Using Endoscopic Approaches in the Surgical Management of Spinal Metastatic Disease

**DOI:** 10.3390/jcm15031093

**Published:** 2026-01-30

**Authors:** Whitney E. Muhlestein, Samuel A. Tenhoeve, Mark A. Mahan

**Affiliations:** 1 Department of Neurosurgery, Clinical Neurosciences Center, University of Utah, Salt Lake City, UT 84132, USA; 2 Spencer Fox Eccles School of Medicine, University of Utah, Salt Lake City, UT 84112, USA

**Keywords:** spine, metastasis, endoscopic spine surgery, minimally invasive

## Abstract

Patients with spinal metastasis often benefit from surgical intervention for debulking to improve neurologic deficits, reduce spinal cord or root compression, and ameliorate pain. Traditionally, large, open fusions have been used to achieve adequate decompression of neural structures. These types of interventions are frequently associated with significant blood loss, prolonged hospitalizations, and increased risk of surgery-related complications, which can delay postoperative chemotherapy and radiation therapies. Endoscopic spine approaches allow access to the spinal cord and nerve roots with minimal soft tissue disruption, which has been shown to reduce risks associated with open surgery in other contexts. Furthermore, the smaller incision, reduced blood loss, ability to position incisions away from radiation fields, and lower risk profile in high-risk patients may provide an effective solution to spinal metastases in appropriately selected cases. Here, we present two cases of spinal metastases successfully managed with spinal endoscopy and recommend the consideration of this approach for similar scenarios.

## 1. Introduction

Spinal metastases occur in a significant proportion of cancer patients, with published incidences of 15–70% [1,2,3,4,5,6,7]. Surgical management may be indicated in cases of metastasis that cause spinal cord compression, mechanical instability, or significant pain. Traditionally, surgical options for spinal metastases have included radical cytoreduction surgery or spinal cord decompression with or without external beam radiation [8]. The development of stereotactic body radiotherapy (SBRT) allows for targeted delivery of higher doses of radiation to lesional tissue but with a requirement of 2–3 mm of space between the spinal cord and metastatic disease to avoid injury to the neural elements. Separation surgery, which aims to create this requisite space, followed by SBRT, has now become the standard of care for most patients with spinal metastases and has been shown to improve local control and overall survival for this population [9,10].

Open surgery for spinal metastases, particularly those involving the vertebral body, often requires transpedicular approaches to gain access to the ventral epidural space. The instability introduced by such an approach necessitates instrumentation, which in turn demands larger incisions, wider bony exposure, and higher intraoperative blood loss, all of which can delay the initiation of SBRT [4,11,12]. Endoscopic spine surgery (ESS) represents an ultra-minimally invasive approach that, through the use of small skin incisions (6–10 mm) and continuous irrigation, allows for surgical debulking of spinal metastases with minimal muscle and bony disruption and high-resolution visualization. Although ESS is relatively well established for degenerative pathologies, it has not gained significant traction for treatment of oncologic disease. It is not indicated in all cases, but in certain circumstances, including purely intracanalicular lesions or recurrent tumors at previously instrumented levels, ESS can be a powerful tool to decrease patient morbidity and time to recovery. Rapidly returning patients to an acceptable quality of life is particularly important in this patient population, given the limited life expectancy associated with spinal metastatic disease.

Here, we describe our experience using an ESS approach for two patients with spinal metastases. We also review the literature comparing open and ESS approaches with respect to perioperative complications, including surgical site infections and blood loss, length of hospitalization, timing of SBRT, and cost.

## 2. Case Illustrations

### 2.1. Case 1

**Figure 1:**
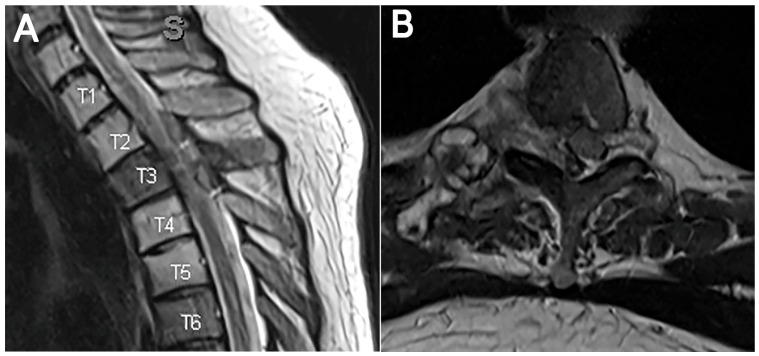
Case 1. Preoperative imaging. (**A**) T2 sagittal imaging demonstrating intradural, extramedullary metastases at T2–T3 and T3–T4. Radiation changes are also visible at T1–T5. (**B**) T2 axial imaging demonstrating associated canal stenosis.

This 34-year-old man with chondroblastic osteosarcoma arising from the right rib with intradural, extramedullary metastases at T2–T3 and T3–T4 with associated canal stenosis presented with upper back pain (Figure 1). After discussion with radiation oncology, the decision was made to resect the lesion sufficiently to facilitate SBRT.

**Figure 2:**
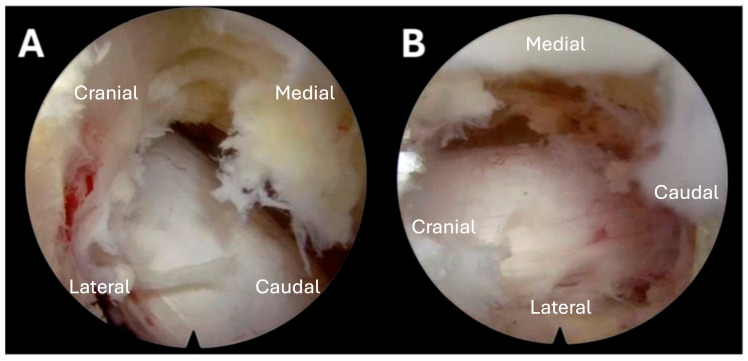
Case 1. Intraoperative endoscopic views after tumor debulking at the T3–T4 level. (**A**) Rostral view demonstrating removal of the right-sided lamina and exposure of the dorsal thecal sac after intracanalicular tumor debulking. (**B**) Caudal view showing decompression across the midline with restoration of cerebrospinal fluid space and visible dural pulsation, confirming adequate separation for SBRT planning.

Because the lesion was entirely intracanalicular, an endoscopic approach was chosen for tumor debulking, beginning with the T2–T3 level. Fluoroscopy was used for the initial approach, and an 18-gauge needle was advanced toward the dorsal lamina at the right T2–T3 laminar-facet junction. Sequential dilation was performed over a wire, the tubular retractor was placed, and the endoscope was introduced. Under direct light-based visualization, the right-sided lamina-facet complex was drilled out from the pars medially to the laminar junction, first removing the lamina of T2 and then the superior articulating process of T3. Once these were completely removed, the tumor was encountered and carefully dissected away from the thecal sac and the T2 exiting nerve root. The T3 lamina was drilled to facilitate tumor removal around the T3 pedicle. Tumor debulking continued until there was clear pulsation of the thecal sac and no evidence of tumor across the midline (Figure 2). The same process was repeated for the T3–T4 level. Blood loss was essentially undetectable because of the tumor type, and there were no obvious intraoperative complications.

**Figure 3:**
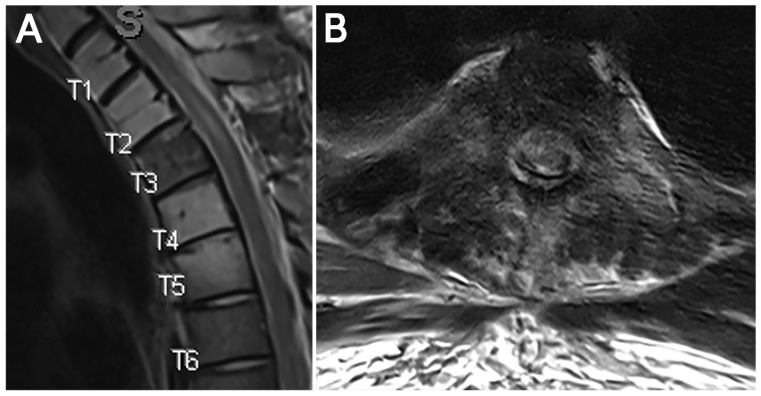
Case 1. Postoperative imaging. (**A**) T2 sagittal imaging and (**B**) axial imaging (somewhat motion degraded) demonstrating decompression of the spinal canal.

Postoperatively, the patient remained at his neurological baseline, and imaging demonstrated no residual tumor in the canal with adequate decompression of the spinal cord (Figure 3) and sufficient margins for radiation. He was cleared for immediate radiation therapy; however, he began SBRT a month after surgery because of an unstable housing situation and continued to improve during a 2-month follow-up period.

### 2.2. Case 2

This 77-year-old man had a history of metastatic renal cell carcinoma with T10 and L4 metastases diagnosed in 2022 for which he underwent external beam radiation therapy only. Several months later, he presented to the emergency department with urinary incontinence, leg numbness, foot drop, and decreased anal sphincter tone. He was found to have a pathologic T10 burst fracture and underwent an open partial T10 corpectomy with T8-L1 posterior fusion.

**Figure 4:**
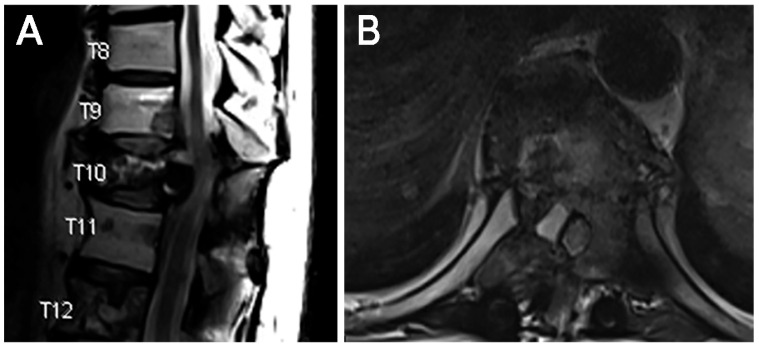
Case 2. Preoperative imaging. (**A**) T2 sagittal and (**B**) axial imaging demonstrating T10 metastatic lesion with associated canal stenosis.

The patient presented again in 2025 with worsening lower extremity paresthesias and numbness from his hips to his knees, impeding his ability to walk. Magnetic resonance imaging demonstrated progression of the known T10 lesion with associated canal stenosis (Figure 4). After discussion with radiation oncology, the decision was made to pursue endoscopic separation surgery via a transpedicular approach to facilitate SBRT.

**Figure 5:**
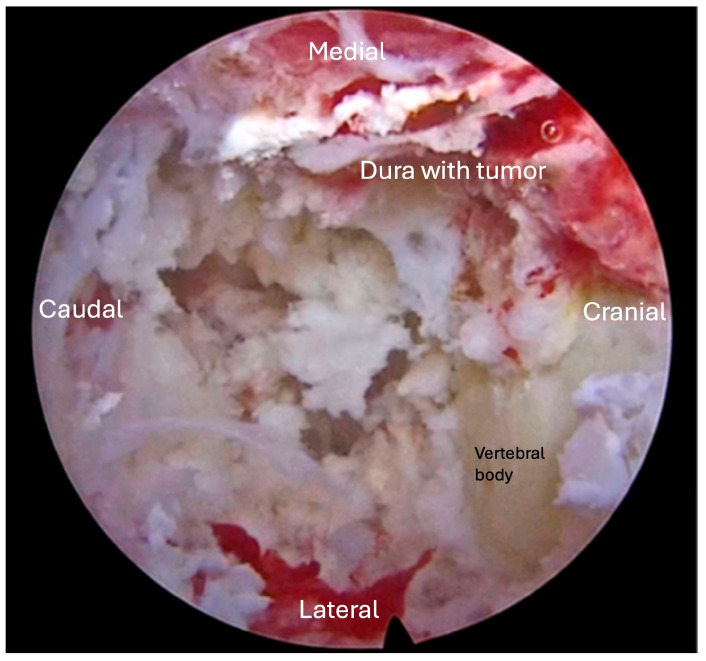
Case 2. Intraoperative endoscopic view during transpedicular decompression at T10. The ventral epidural space is accessed through a unilateral transpedicular corridor, allowing debulking of the posterior vertebral body tumor beneath the thecal sac. Clear dural pulsation is visible at the superior aspect of the field, indicating adequate spinal cord decompression without the need for revision instrumentation.

Under fluoroscopic guidance, the tip of an 18-gauge needle was placed at the junction of the pedicle and the T10 transverse process. After successful dilation, a tubular retractor was placed, and an endoscope was introduced. Under direct visualization, the T10 rib head, transverse process, and pedicle were drilled down to allow for spinal cord decompression. The dorsal T10 vertebral body was debulked beneath the thecal sac until there was clear pulsation of the dura (Figure 5). Because aggressive unilateral debulking was performed, bilateral decompression was not pursued. Blood loss was 30 mL, and there were no obvious intraoperative complications.

**Figure 6:**
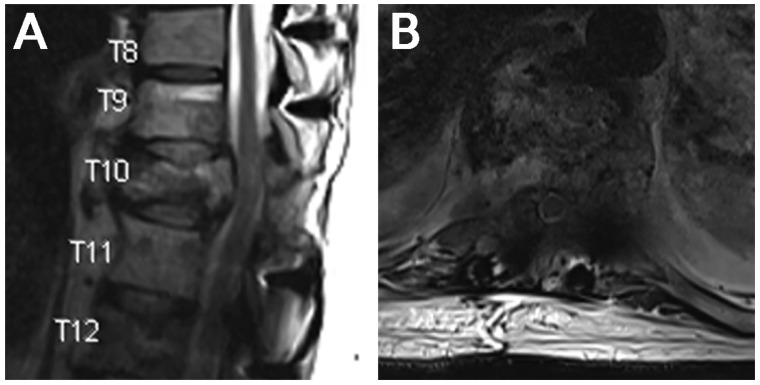
Case 2. Postoperative imaging. (**A**) T2 sagittal and (**B**) axial imaging demonstrating improved canal caliber.

Postoperatively, the patient remained neurologically at his baseline. Postoperative magnetic resonance imaging demonstrated improved canal caliber (Figure 6). He underwent SBRT 3 weeks later and had no associated wound complications. Over a 5-month follow-up period, the patient continued to improve.

## 3. Discussion

ESS is a powerful tool that can improve outcomes for patients with spinal metastases by avoiding instrumentation, lowering intraoperative blood loss, decreasing the risk of postoperative wound infection, minimizing hospital length of stay, and expediting the initiation of SBRT.

### 3.1. Avoidance of Instrumentation

As illustrated in these cases, ESS provides an opportunity for noninstrumented debulking of spinal metastases, particularly in cases of intracanalicular lesions or revision surgery. In cases in which access to the ventral epidural space is required, the ability to access a transforaminal corridor and the high-magnification, high-resolution visualization afforded by ESS help minimize the amount of pedicle and facet joint drilling required. Avoidance of instrumented fusion limits blood loss, postoperative pain, perioperative complications, duration of surgery, wound complications, need for postoperative intensive care, length of hospitalization, and length of time to initiation of SBRT [13,14]. In cases where patients have had a prior spinal fusion, an ESS approach may also obviate the need for revision instrumentation.

### 3.2. Blood Loss

Estimated blood loss exceeding 500 mL is strongly associated with major postoperative complications in patients with spinal metastases, significantly increasing the likelihood of cardiovascular events, pulmonary insufficiency, and stroke [15]. Many patients with metastatic cancer already have diminished cardiopulmonary reserve, chronic anemia, malnutrition, or myelosuppression from systemic therapy, rendering them less able to tolerate even moderate intraoperative hemorrhage. High blood loss may also be associated with a greater risk of surgical site infection, because blood clots can act as a breeding ground for bacteria.

ESS limits blood loss by minimizing local tissue disruption, using continuous irrigation, which provides a tamponade effect, and allowing for hemostasis under direct visualization [16]. In a systematic review of 61 patients who underwent ESS for spine metastasis, the mean estimated blood loss was only 49 mL [17]. In these cases, we report a mean estimated blood loss of 15 mL.

Open separation surgery may be prone to higher rates of hemorrhage because wide exposure is inherent to access the ventral epidural space, often necessitating violation of the paraspinal musculature and the valveless epidural plexus. Further, tumor friability, prior irradiation, and tumor-induced neovascularity may further amplify bleeding risk. Published series reflect this reality, with the mean estimated blood loss for open separation surgery ranging from 700 mL to 2000 mL [18].

### 3.3. Wound Infection

Surgical site infections remain one of the most consequential postoperative complications in the management of spinal metastases. These infections contribute not only to increased readmission rates but also to prolonged hospital stays, higher healthcare costs, and greater patient discomfort, impaired functional recovery, and delays in adjuvant therapies [19,20]. Patients with spinal metastases are at particularly high risk of wound infection for a myriad of reasons, including neurologic deficits, which often result in decreased mobilization; systemic adjuvant therapies, which weaken immunity; and prior surgeries or other locations of metastases. In a meta-analysis of over 2000 patients treated surgically for spinal metastases, surgical site infection was the most reported complication, present in 6.5% of cases [21].

ESS approaches reduce the risk of surgical site infection through several mechanisms. The markedly smaller incision minimizes dermal and subcutaneous disruption, while serial dilation preserves muscle fibers rather than stripping them from their attachments. The absence of a large dead space may reduce the potential for seroma and hematoma formation. Continuous irrigation maintains a clear operative field and circulates out accumulated biomass (including blood), removing potential sites for infection [22]. These benefits have borne out in propensity score-matched analyses: Mahan and colleagues [22] demonstrated that patients who underwent ESS had a 0.001% risk of surgical site infection compared with a 1.1% risk in patients who underwent non-ESS, representing a 16-fold risk reduction.

### 3.4. Length of Stay

Prolonged hospital stays increase the risk of nosocomial infection, venous thromboembolism, deconditioning, and delays in initiating adjuvant therapies [23,24]. They also carry substantial implications for healthcare utilization and cost in a population that often has limited physiologic reserve and complex care needs [25]. ESS is typically associated with relatively short hospitalizations, especially compared with open operations. In a systematic review of patients who underwent endoscopic approaches for spinal tumors (including intradural lesions), Sofoluke and colleagues [17] reported a mean length of stay of 55.2 h. By contrast, Pennington et al. [18] reported mean lengths of stay ranging from 9.5 to 24 days for patients undergoing open separation surgery. The cases presented in this study had longer lengths of stay and may indicate that preoperative functional limitations or social factors are primary drivers of length of stay. One patient remained hospitalized because of baseline neurologic deficits and housing instability rather than complications from the procedure itself.

### 3.5. Minimally Invasive Approaches in Spine Oncology

Although the bulk of ESS literature has focused on degenerative pathology [26], a growing body of evidence supports the broader applicability of minimally invasive techniques across diverse spinal conditions, including spinal oncology [27,28,29,30,31]. The role of MIS surgery in oncologic care has expanded in parallel with advances in radiation delivery and a fundamental shift in surgical objectives. As recently outlined by Newman et al., contemporary management of metastatic spinal disease has grown to prioritize reconstitution of the thacal sac to permit delivery of ablative SBRT rather than extensive tumor cytoreduction [30]. Wihtin this framework, minimally invasive spine surgery techniques have gained greater adoption due to their ability to achieve adequate decompression and stabilization while minimizing surgical morbiditiy, blood loss, and delays in adjuvant therapy. Importantly, this evolution has opened the door to ultra-minimally invasive strategies, including ESS and robotic-assisted approaches, which may further reduce the surgical footprint in carefully selected patients [32].

### 3.6. Early SBRT

Radiotherapy is known to have a negative impact on wound healing and bony fusion after surgery. Fibroblasts, which play a critical role in laying down collagen and extracellular matrix components in the first few days after closure of a surgical wound, are particularly sensitive to the effects of radiation, and their derangement after radiation exposure may explain deficits in wound healing after early radiotherapy [33].

These considerations must be balanced with the clear association of rapid initiation of SBRT after surgery and overall survival for patients with spinal metastases [34,35]. In a study of 89 patients, Gong and colleagues [7] demonstrated that the risk of local progression in patients who underwent radiotherapy within 1 month of surgery was only 1.2% but jumped to 24.1% and 45.1% in patients treated at 3 and 6 months, respectively. They also showed that overall survival was inversely proportional to rates of local progression.

ESS uses incisions smaller than 1 cm and serial dilation to minimize trauma to deeper tissues, thereby reducing the amount of tissue at risk for radiation-induced injury. Postoperative measurements of C-reactive protein and creatine phosphokinase demonstrate a diminished systemic inflammatory response, underscoring how the endoscopic approach limits tissue disruption. Consequently, experts suggest that SBRT can be safely initiated within 2–3 days after minimally invasive surgery, compared with 2 weeks for open approaches [36].

In a single-center study, Dugan et al. [8] found that more than 50% of patients who underwent separation surgery did not receive radiotherapy within 3 months, despite the fact that at least 80% of these patients were considered to be good candidates for radiation. A portion of these patients failed to initiate radiation because of loss to follow-up, as was the case for one of the patients we describe. Minimizing the time between surgery and SBRT may be particularly important in limiting the chance that patients become lost to the healthcare system and thus fail to benefit from early radiation treatment.

### 3.7. Overall Costs

Stewardship of limited healthcare resources is the responsibility of all physicians. ESS does require important upfront capital investments, including an endoscope, endoscopy tower, and endoscope-specific tools, some of which are single use. Using propensity score-matching, Findlay et al. [37] found that ESS procedures were associated with higher overall hospital costs than open spine procedures (115.1% vs. 100%), but lower mean pharmacy and laboratory costs. However, patients who underwent ESS also had shorter hospital stays (0.7 vs. 1.4 days) and fewer perioperative complications (3.1% vs. 7.9%). In a similar study, Leyendecker et al. [38] showed that ESS was associated with a fourfold reduction in hospital readmission compared with open procedures. Supply costs thus appear to be the primary driver of the higher expense of ESS compared with open surgery and represent an opportunity for improvement and optimization.

### 3.8. Limitations of ESS

There are important limitations associated with the use of ESS for spinal metastatic disease. There is a steep learning curve to the safe and efficacious use of ESS, particularly for oncologic cases, which are often more technically demanding the degenerative spine pathologies. Additionally, a limited operative corridor can make hemostasis more difficult in cases of hypervascular tumors. The use of preoperative embolization, continuous irrigation containing epinephrine, meticulous coagulation with bipolar cautery, careful patient positioning to avoid venous congestion, and strict blood pressure parameters can help mitigate these challenges. Docking of the endoscope working channel on compromised, tumor-laden bone should also be performed with caution.

A concern with ESS in oncologic surgery is the risk of tumor cell spread through ongoing irrigation. Similar worries have been raised in arthroscopy and endoscopic tumor removal across different surgical fields [39]. However, evidence suggests that continuous irrigation may dilute and flush out free tumor cells rather than cause implantation, especially when combined with constant suction and controlled outflow, although multiple factors are involved [40]. Although the literature is limited, it seems reasonable to hypothesize that the sealed working channel, continuous suction, and shorter surgery times associated with ESS could protect against tumor cell spread. Still, high-quality comparative oncologic data are scarce, highlighting the importance of careful patient selection and precise surgical technique.

Although ESS may provide perioperative benefits, its long-term oncologic outcomes have not yet been definitively compared with open decompression. In the current approach to spinal metastases, durable local control appears to be achieved primarily through effective separation and subsequent SBRT, rather than extensive surgical removal. Therefore, ESS can be seen as a way to obtain sufficient decompression with less morbidity, rather than as a method for oncologic resection. Lastly, ESS may not be appropriate for patients with intracanalicular or ventral epidural disease without gross instability. Patients with extensive vertebral body destruction, high-grade instability, or hypervascular tumors may be less suitable candidates, and therefore, proper patient selection and the development of standardized metrics are paramount and deserve extended investigation.

## 4. Future Perspectives

Of important note, artificial intelligence (AI) is increasingly poised to influence surgical decision-making in spine oncology. Predictive models integrating radiographic features, tumor histology, biomechanical stability, and patient-specific risk factors may help identify patients most likely to benefit from minimally invasive or endoscopic approaches [41]. Machine learning algorithms may also aid in estimating surgical morbidity, anticipated recovery time, and optimal timing of adjuvant therapies such as SBRT [41]. As these tools mature, AI-driven decision support systems may help refine patient selection for ESS, ensuring oncologic goals are met while minimizing procedural risk.

## 5. Conclusions

ESS can be used safely and effectively to treat patients with spinal metastatic disease. By minimizing the risk of perioperative complications, including wound breakdown, surgical site infection, and excessive blood loss, ESS may reduce hospitalization length and expedite SBRT initiation, thereby improving long-term outcomes for these patients.

## Data Availability

No new data were created or analyzed in this study. Data sharing is not applicable to this article.

## Abbreviations

The following abbreviations are used in this manuscript:

SBRT	Stereotactic body radiotherapy
ESS	Endoscopic spine surgery
